# Demographic characteristics and repeat overdose risk factors in 601 patients with intentional drug overdose in the emergency department: a cross-sectional study

**DOI:** 10.3389/fpsyt.2026.1763816

**Published:** 2026-02-05

**Authors:** Shumin Rong, Yonghui Wan, Han Zhou, Chun Zhang, Meiling Fan, Jie Hao, Xiaojie Yan

**Affiliations:** 1 Emergency Department, Renmin Hospital of Wuhan University, Wuhan, Hubei, China; 2 Department of Nursing, Renmin Hospital of Wuhan University, Wuhan, Hubei, China; 3 Department of Oncology, Renmin Hospital of Wuhan University, Wuhan, Hubei, China; 4 Department of Gynecology, Renmin Hospital of Wuhan University, Wuhan, Hubei, China; 5 Department of Traditional Chinese Medicine, Wudong Hospital of Wuhan, Wuhan, Hubei, China

**Keywords:** emergency department, emergency nursing, intentional drug overdose, prevention strategies, recurrence, risk factors, self-injurious behavior

## Abstract

**Objective:**

To examine the demographic and clinical characteristics of patients with intentional drug overdose (IDO) presenting to the emergency department and to identify risk factors associated with repeated IDO, thereby providing an evidence-based foundation for emergency nursing interventions and prevention strategies.

**Methods:**

A retrospective cross-sectional study was conducted using emergency department medical records of 601 patients with intentional drug overdose (IDO) (ICD-10 codes: T36–T50) who presented to a tertiary hospital in Wuhan, China, between January 2023 and December 2024. Demographic characteristics, seasonal distribution, substance categories, psychiatric history, alcohol use, and prior self-harm were collected. Descriptive statistics, chi-square tests, and multivariable logistic regression analyses were performed.

**Results:**

The majority of patients were female (70.4%) and adolescents or young adults aged 10–29 years (72.2%), with a median age of 22 years. Overall, IDO cases demonstrated seasonal peaks in autumn and spring, accounting for 61.6% of presentations. Age-stratified analyses revealed significant seasonal variation (χ² = 27.064, *p* = 0.008), with adolescents and young adults showing a pronounced autumn peak (both >35%), whereas middle-aged adults (45–59 years) exhibited a relative summer peak. Medication-related poisoning accounted for 96.7% of cases, primarily involving antipsychotics and sedative–hypnotics, while pesticide poisoning comprised 3.3%. A history of prior self-harm was independently associated with repeated IDO (OR = 26.66, 95% CI: 11.17–63.61, *p* < 0.001). Conclusion: Patients presenting with IDO in the emergency department were predominantly adolescents and young adults, with a higher proportion of females. Overall presentations peaked in autumn and spring, largely driven by younger age groups. A prior history of self-harm was independently associated with repeated IDO. Emergency nurses should routinely assess self-harm history, prioritize early identification and intervention among high-risk adolescents and young adults, and strengthen integrated “emergency–psychiatry–community” care pathways to reduce recurrence.

## 1 Introduction

Suicide is a serious global public health problem, with approximately 727,000 people dying by suicide each year and many more attempting it, according to the World Health Organization (WHO). Suicide can occur across all stages of life. In 2021, it ranked as the third leading cause of death worldwide among individuals aged 15–29 years (1). One of the most common forms of suicide-related behavior is self-harm, which refers to intentional self-poisoning or self-injury, regardless of intent or outcome. It can manifest in various forms, including IDO, ingestion of harmful substances, cutting, burning, or hitting oneself (2). Self-cutting is the predominant form in community settings, whereas self-poisoning is the most frequent presentation in hospital settings worldwide (2, 3). IDO is particularly common in emergency departments (EDs). Emergency nurses, as the first-line caregivers who come into contact with these patients, are primarily responsible for monitoring vital signs and conducting risk assessments. They also play a central role in psychological crisis recognition, emotional support, and health education. Timely and effective nursing interventions can substantially reduce the risk of repeated IDO and improve patient outcomes. Previous studies, both domestic and international, have reported seasonal patterns in IDO-related consultations (4), sex differences (5), and population-specific characteristics (6, 7). Repeated self-harm refers to situations where an individual engages in non-accidental self-harm or self-injurious behavior on multiple occasions (2). Such behaviors are strongly associated with future suicide risk, particularly among adolescents and young adults (8). Frequent or repeated self-harm is also a major risk factor for suicide mortality (9). Although several international studies have focused on repeated self-harm, there are limited data on IDO in emergency care settings in China. Domestic studies have documented differences in poisoning types and patient characteristics across regions—for instance, pesticide poisoning is more prevalent in rural areas, whereas drug poisoning predominates in urban areas (10, 11). Furthermore, the incidence of poisoning has been increasing annually. However, most existing studies combine intentional and accidental poisoning, are limited to single centers, and lack detailed exploration of IDO-specific and repeated IDO risk factors in emergency settings. Therefore, this study aimed to analyze the demographic and psychosocial characteristics of 601 IDO patients treated in the ED of a tertiary hospital in Wuhan, China, using real-world data. Specifically, we sought to examine the seasonal distribution of IDO across different age groups and identify the risk factors associated with repeated IDO. The findings of this study are expected to provide an evidence-based foundation for emergency nurses to conduct risk screening and implement targeted clinical interventions for high-risk populations. Moreover, the results may contribute to the development of an integrated “emergency–psychiatry–community” continuum of care model and serve as a reference for designing more effective prevention strategies.

## 2 Materials and methods

### 2.1 Study design and participants

This retrospective cross-sectional study using emergency department medical records included patients with IDO who presented to the Emergency Department (ED) of a tertiary general hospital in Wuhan, China, between January 2023 and December 2024. All eligible cases were identified through the hospital’s Electronic Medical Record (EMR) system. Patients diagnosed with drug poisoning or overdose were identified and screened from the ED database using the International Classification of Diseases, 10th Revision (ICD-10) coding system (codes T36–T50).

Inclusion criteria. 1. Intentional drug overdose. 2. Presentation as an acute medical emergency.

Exclusion criteria. 1. Unintentional drug overdose (e.g., accidental ingestion or adverse drug reaction). 2. Repeated visits for the same overdose event.

Because the study was retrospective, anonymized, and non-interventional, the requirement for informed consent was waived, and the study protocol was approved by the Clinical Research Ethics Committee (approval number: WDRM2025-K129).

### 2.2 Data collection

After obtaining raw data from the EMR system, the researchers developed a customized data extraction form based on the study objectives and the variables required for analysis. All medical records were systematically reviewed, and the relevant variables were extracted and recorded using this standardized form. According to the established inclusion and exclusion criteria, a total of 601 eligible cases were included in the final dataset.

#### 2.2.1 Data organization and analysis

The collected variables included demographic characteristics (sex, age, season of visit), clinical information (history of psychiatric disorders, history of chronic diseases, alcohol use, and history of self-harm), and toxicological characteristics (substance categories and whether the IDO was a repeated event). Age groups were categorized according to the World Health Organization (WHO) and Chinese national criteria as follows: 10–19 years (adolescents), 20–29 years (young adults), 30–44 years (adults), 45–59 years (middle-aged adults), and 60–98 years (older adults). Seasons were defined based on the month of admission: spring (March–May), summer (June–August), autumn (September–November), and winter (December–February) (4). Drug classification was based on the Anatomical Therapeutic Chemical (ATC) Classification System, differentiating between pharmaceutical agents for therapeutic purposes and chemical or biological agents used in agriculture (e.g., pesticides, herbicides). Details are available in the ATC/DDD Index and Guidelines (12). Psychiatric history was extracted from the EMR at the time of emergency department presentation. This variable was defined as a documented history of psychiatric disorders prior to the index overdose, based on physician documentation during clinical assessment. Information was obtained through a combination of patient or family self-report, review of prior medical records, and routine inquiry about past psychiatric diagnoses and treatment history.

Psychiatric history included any previous diagnosis of mental disorders (e.g., depressive disorders, anxiety disorders, bipolar disorder, schizophrenia or other psychotic disorders) or prior contact with psychiatric services. Current psychiatric medication use was recorded when available. Importantly, history of psychiatric disorders was treated as a separate variable from history of self-harm, which was analyzed independently in the study. Alcohol use was assessed based on documentation in the EMR, including patient or family report of alcohol consumption prior to the overdose event or a known history of alcohol use noted during clinical assessment. Information on other drugs of abuse (e.g., illicit substances) was not systematically available in the medical records and was therefore not included as an independent variable in the analysis. Sex was included as a binary variable in the regression analyses, with males serving as the reference category. Age was included as a continuous variable in the multivariable logistic regression model regardless of statistical significance. All variables were coded using standardized procedures prior to statistical analysis.

Statistical analyses were performed using IBM SPSS Statistics version 30 (IBM Corp., Armonk, NY, USA), with a two-sided *p* value < 0.05 considered statistically significant.

### 2.3 Statistical analysis

Dual data entry and verification were performed to ensure accuracy. Any ambiguous or missing information in the EMR was rechecked and confirmed by a third independent researcher. The inclusion and exclusion criteria were strictly applied to minimize selection bias. Statistical analyses were conducted using IBM SPSS Statistics, version 30.0 (IBM Corp., Armonk, NY, USA). Continuous variables were assessed for distributional characteristics and, as they did not follow a normal distribution, were summarized as medians with interquartile ranges (IQRs). Categorical variables were summarized as frequencies and percentages. Differences between two categorical variables were tested using the chi-square test (*χ²* test). Univariate analyses were first performed using chi-square tests to screen potential factors associated with repeated IDO. Variables with a p value ≤ 0.10 in univariate analyses were considered candidates for multivariable logistic regression. In addition, age and sex were included in the multivariable model *a priori*, regardless of their statistical significance, based on clinical relevance. Multivariable logistic regression analysis was subsequently conducted to identify factors independently associated with repeated IDO. All statistical tests were two-sided, and a *p* value < 0.05 was considered statistically significant.

### 2.4 Calculation of sample size

The significance level was set at α = 0.05, corresponding to *Z*_α/2_ = 1.96.

Assuming the maximum conservative estimate of *P* = 0.5 and a permissible error (δ) of 0.04, the required sample size was calculated using the formula:


$$n=\frac{Z_{\alpha /2}^{2}P(1-P)}{\delta^{2}}$$

By substituting these values into the equation, the minimum required sample size was 600.25, which was rounded up to 601 cases. Therefore, the inclusion of 601 patients in this study met the calculated sample size requirement.

## 3 Results

### 3.1 Demographic and clinical characteristics

The demographic and clinical characteristics of patients with IDO are summarized in (**Table 1**) Reference source not found. and stratified by sex. A total of 601 patients were included in the analysis. Most of the patients were hospitalized or remained in the hospital after emergency treatment. Some recovered and were discharged, a few were transferred to psychiatric outpatient clinics or other hospitals for further treatment, or left against medical advice. One outpatient death was recorded.

### 3.2 Distribution of poisoned substance types

Among the 601 patients, medication-related poisoning accounted for the majority of cases (96.7%). The most frequently involved medication classes were antipsychotic agents (ATC code N05A), particularly lithium carbonate and quetiapine, **sedative–**hypnotics (ATC code N05C), mainly eszopiclone and zopiclone. Pesticide-related poisoning constituted 3.33% of the total, including diquat (3 cases, 15%), glyphosate (2 cases, 10%), bromadiolone (3 cases, 15%), and pyrethroids (2 cases, 10%), among others.

A wide range of medication categories was identified among patients with IDO. Antipsychotics and sedative–hypnotics were the most frequently involved substances, whereas other medication categories—including antidepressants, antibiotics, cardiovascular agents, and miscellaneous drugs—were observed at substantially lower frequencies and are summarized descriptively. The detailed distribution of medication categories is provided in **Supplementary Table S1**.

### 3.3 Differences in age grouping and seasonal distribution of IDO patients

Overall, IDO cases demonstrated seasonal peaks in autumn and spring. Age-stratified analyses revealed significant differences in seasonal distribution across age groups. Adolescents (10–19 years) and young adults (20–29 years) showed a pronounced autumn peak, with more than 35% of cases occurring during this season. In contrast, middle-aged adults (45–59 years) exhibited a different seasonal pattern, with the highest proportion of cases observed in summer (35.7%). Chi-square analysis confirmed a significant association between age group and seasonal distribution of IDO cases (χ² = 27.064, *p* = 0.008), with a small effect size (Cramer’s V = 0.123; **Table 2**). By comparison, no significant difference in seasonal distribution was observed between males and females (*p* = 0.909; **Table 1**).

**Table 1:** Demographic and clinical characteristics of patients with IDO in the emergency department, stratified by sex.

Variable	Total (*n* = 601)	Male (*n* = 178, 29. 6%)	Female (*n* = 423, 70.4%)	*P-value*
Age (years, median [IQR])	22 (19–31)	22 (19-29)	22 (19-32)	0.432
Age group				0.599
Adolescents (10–19 years)	198	63 (31.8)	135 (68.2)	
Young adults (20–29 years)	236	72 (30.5)	164 (69.5)	
Adults (30–44 years)	81	22 (27.2)	59 (72.8)	
Middle-aged (45–59 years)	28	5 (17.9)	23 (82.1)	
Older adults (60–98 years)	58	16 (27.6)	42 (72.4)	
Season (*n*, %)				0909
Spring	181	50 (27.6)	131 (72.4)	
Summer	115	36 (31.3)	79 (68.7)	
Autumn	189	57 (30.2)	132 (69.8)	
Winter	116	35 (29.6)	81 (69.8)	
Repeated IDO	31	9 (29.0)	22 (71.0)	0.942
Chronic disease history	103	27 (26.2)	76 (73.8)	0.406
Psychiatric history	434	129 (29.7)	305 (70.3)	0.927
Alcohol use	46	14 (30.4)	32 (69.6)	0.899
History of self-harm	76	21 (27.6)	55 (72.4)	0.685

Values are expressed as n (%) unless otherwise indicated. Median (interquartile range, IQR) was used for continuous variables due to skewed distribution. P-values were calculated using the Mann–Whitney U test for continuous variables and the χ² test for categorical variables.

**Table 2:** Differences in seasonal distribution among age groups of patients with IDO in the emergency department.

Age group (years)	Spring, *n* (%)	Summer, *n* (%)	Autumn, *n* (%)	Winter, *n* (%)	*χ² (df = 12)*	*P-*value	*Effect size (Cramer’s V)*
Adolescents (10–19)	53 (26.8)	37 (18.7)	70 (35.4)	38 (19.2)			
Young adults (20–29)	81 (34.3)	41 (17.4)	83 (35.2)	31 (13.1)			
Adults (30–44)	24 (29.6)	17 (21.0)	18 (22.2)	22 (27.2)			
Middle-aged (45–59 years)	5 (17.9)	10 (35.7)	5 (17.9)	8 (28.6)			
Older adults (60–98 years)	18 (31.0)	10 (17.2)	13 (22.4)	17 (29.3)			
Total	181	115	189	116	27.064	0.008	0.123

All expected frequencies were > 5 (minimum expected value = 5.36). According to Cramer’s V interpretation, 0.1 ≤ V < 0.3 indicates a small effect size.

### 3.4 Analysis of factors associated with repeated IDO

In the multivariable logistic regression analysis, a history of self-harm was independently associated with repeated IDO, with an adjusted odds ratio (OR) of 26.66 (95% CI: 11.17–63.61, *p* < 0.001). Age and sex were not independently associated with repeated IDO, nor was psychiatric history after adjustment for covariates (OR = 1.49, 95% CI: 0.39–5.75, *p* = 0.563; **Table 3**). The final model demonstrated an adequate goodness-of-fit, as indicated by the Hosmer–Lemeshow test (χ² = 9.30, df = 8, *p* = 0.318). .

**Table 3:** Multivariable logistic regression analysis of factors associated with repeated IDO.

Variable	*B*	*SE*	*Wald χ²*	*p-*value	OR (95% *CI*)
Age (per 1-year increase)	0.005	0.014	0.117	0.733	1.01 (0.98–1.03)
Sex (female vs male)	−0.042	0.455	0.009	0.926	0.96 (0.39–2.34)
Psychiatric history (Yes = 1, No = 0)	0.399	0.689	0.335	0.563	1.49 (0.39–5.75)
History of self-harm (Yes = 1, No = 0)	3.283	0.444	54.755	<0.001	26.66(11.17–63.61)

The dependent variable was repeat IDO (1, yes; 0, no). Age and sex were included in the multivariable logistic regression model *a priori*, regardless of their statistical significance. Other variables with *p* ≤ 0.10 in the univariable analysis were additionally included. The alcohol use variable was excluded due to complete separation (no cases in the repeat IDO group), which prevented stable estimation. OR, odds ratio; CI, confidence interval. A two-sided *p* < 0.05 was considered statistically significant.

In univariable analyses, history of self-harm, psychiatric history, and alcohol use were included in the multivariable logistic regression analysis. However, alcohol use was excluded from the final analysis because no cases of alcohol use were observed among patients with repeated IDO, resulting in complete separation and preventing stable parameter estimation.

## 4 Discussion

This study examined 601 patients with IDO presenting to the emergency department between 2023 and 2024, providing a comprehensive overview of demographic characteristics, medical history, substance ingestion patterns, age- and season-specific distributions, and factors associated with repeated IDO. From the perspective of the multilevel determinants of health, self-harm behavior results from the interplay of factors operating at different levels, including individual psychological and physiological attributes (downstream), social and familial support systems (midstream), and broader social, economic, and structural conditions (upstream) (2). Population-level evidence has demonstrated that structural disadvantages—such as poverty, income inequality, and lower Human Development Index (HDI)—are associated with increased overdose mortality, underscoring the influence of upstream determinants on self-harm outcomes (13). These upstream influences highlight the importance of considering not only individual clinical risk factors but also broader contextual determinants when interpreting IDO patterns. Within this multilevel context, the present findings indicate that IDO presentations in the emergency setting are more common among females and younger individuals, and that temporal patterns vary across the year. However, when factors associated with repeated IDO were examined, a history of prior self-harm emerged as the only variable independently associated with repeated IDO in the multivariable analysis, whereas demographic characteristics alone were not sufficient to explain repetition risk. This distinction highlights that demographic and temporal factors are primarily associated with the initial presentation of IDO, whereas a history of prior self-harm is more relevant for identifying patients at increased risk of repeated IDO.

### 4.1 Demographic characteristics and gender differences

In this study, females accounted for more than two-thirds of patients presenting with IDO, a pattern that was consistently observed across all age groups. However, it should be noted that sex-stratified comparisons did not reveal statistically significant differences between males and females, indicating that sex was not an independent differentiating factor in the demographic or clinical characteristics examined.

Importantly, the predominance of female patients reflects a descriptive pattern of IDO presentation in the emergency department rather than a statistically significant sex-based difference. Similar distributions have been reported in previous epidemiological studies, which consistently show higher rates of self-harm among women (14–17), despite higher suicide mortality rates among men (15). Such sex-related patterns have been commonly attributed to differences in help-seeking behavior (18), method selection, and psychosocial vulnerability (19) rather than differences in suicidal intent alone. In particular, prior studies suggest that females tend to have greater contact with healthcare services and may be more willing to disclose psychological distress or seek medical help following self-harm (20), resulting in higher detection and reporting rates in clinical settings. These behavioral and healthcare utilization patterns may partly explain the overrepresentation of female patients among emergency department–based IDO samples, without implying a statistically significant sex-based difference in underlying risk.

### 4.2 Age distribution and seasonal variation of IDO

IDO in this study was predominantly observed among adolescents and young adults, who together accounted for more than two-thirds of all emergency department presentations. This age concentration is consistent with prior studies from China and other countries, which have identified younger populations as a high-risk group for self-harm–related behaviors (7, 21–24). Developmental vulnerability, emotional dysregulation, and heightened sensitivity to psychosocial stressors during adolescence and early adulthood are commonly proposed mechanisms underlying this pattern (25–27).

At the aggregate level, IDO presentations demonstrated seasonal peaks in autumn and spring, accounting for 61.6% of cases. However, age-stratified analyses revealed substantial heterogeneity in seasonal patterns across age groups. Adolescents and young adults exhibited a pronounced autumn peak, with more than one-third of cases occurring during this season, whereas middle-aged adults showed a different temporal distribution, with a relative increase in summer presentations. These findings indicate that the observed seasonal pattern is largely driven by younger individuals and shaped by age-specific psychological vulnerability and psychosocial stressors, rather than reflecting a uniform seasonal effect across all populations. The autumn predominance among adolescents and young adults may be attributable to several age-specific stressors. In the Chinese context, autumn coincides with the beginning of a new academic year, which is often accompanied by increased academic pressure, social adjustment challenges, and heightened expectations related to educational performance. Similar associations between school-related stress and increased self-harm behaviors during academic terms have been reported in both Asian and Western populations (28–31). In addition, seasonal affective changes, including fluctuations in mood and sleep patterns, may disproportionately affect younger individuals with underlying emotional vulnerability, thereby increasing the risk of self-harm behaviors during this period (32–34). In contrast, the seasonal pattern observed among middle-aged adults differed from that of younger age groups, with a relative increase in summer presentations. This divergence suggests that the drivers of IDO may vary across the life course. Middle adulthood is often characterized by cumulative work-related stress, financial responsibilities, and caregiving burdens, which may interact with environmental factors such as high temperatures, sleep disturbance, and occupational strain during summer months. Although the number of middle-aged cases in this study was relatively small, the observed heterogeneity highlights that seasonal variation in IDO is not monolithic and should be interpreted within an age-specific framework.

Importantly, the study period (2023–2024) corresponds to the post–COVID-19 era, during which multiple studies have reported a sustained increase in psychological distress, depressive symptoms, and self-harm behaviors, particularly among adolescents and young adults (35–37). Prolonged social isolation, disruption of daily routines, academic uncertainty, and reduced access to mental health services during the pandemic may have exerted delayed effects on youth mental health, contributing to a rebound in self-harm–related emergency presentations after the acute phase of the pandemic. Although causal inferences cannot be drawn from the present data, the observed age- and season-specific patterns should be interpreted against this broader post-pandemic backdrop.

From a clinical and nursing perspective, these findings underscore the importance of age-sensitive and season-aware risk assessment in the emergency department. Adolescents and young adults represent a particularly vulnerable group during autumn, and targeted screening for psychological distress and self-harm risk during this period may facilitate earlier identification and intervention. Recognizing that seasonal trends are shaped by age-specific psychosocial contexts rather than uniform temporal effects can support more precise prevention strategies and inform the allocation of mental health resources within emergency care settings.

### 4.3 Patterns of substance ingestion

In this study, medication-related poisoning accounted for 96.7% of cases, whereas pesticide-related poisoning represented only 3.3%, indicating that IDO in this emergency department–based study was predominantly medication-related. This pattern is consistent with an urban, hospital-based sample in which prescription medications are readily available within households, particularly among individuals with prior healthcare contact and psychiatric comorbidity.

When comparing our findings with reports from Europe and the United States—where acetaminophen (paracetamol) and non-steroidal anti-inflammatory drugs (NSAIDs) are frequently implicated in self-harm—several contextual factors may explain their lower prominence in our study. One important factor relates to differences in medication availability and purchasing practices. In many Western settings, acetaminophen/NSAIDs are widely accessible as over-the-counter products and may be purchased in large quantities, facilitating impulsive ingestion (38, 39). In contrast, the composition of medications stored at home and patterns of stockpiling may differ in China, and patients with psychiatric conditions may have greater access to sedative–hypnotics, antipsychotics, and other psychotropic prescriptions, which were more prominent in our sample. In addition, differences in reporting and classification may contribute to the apparent discrepancy. In our analysis, NSAIDs were identified in the dataset but accounted for a relatively small proportion of cases. As a result, they did not emerge as dominant categories when results were summarized at the medication-class level. The detailed distribution of all medication categories is provided in **Supplementary Table S1**.

From a prevention standpoint, these findings support the importance of safe medication storage and limiting access to high-risk drugs in vulnerable individuals and households. Strengthening prescription monitoring and early-warning strategies within healthcare systems may help identify potentially inappropriate prescribing or accumulation of high-risk medications (40). Family engagement and health education may further improve medication safety at home and reduce inappropriate use (41). At the policy level, structured prescription surveillance programs, such as the U.S. Prescription Drug Monitoring Program (PDMP), may serve as a reference for strengthening system-level oversight of prescription medications (42).

Finally, although pesticide poisoning was uncommon in this urban emergency study, the presence of these cases suggests that rural or peri-urban exposure may still be relevant, even within urban emergency settings. Continued efforts to improve pesticide regulation, safe storage, and public safety education are warranted, particularly for populations with easier access to agricultural chemicals.

### 4.4 Risk factors for repeat IDO and psychosocial determinants

In the present study, a history of prior self-harm was significantly associated with repeated IDO. This finding is consistent with a broad body of existing research, which has repeatedly reported a close association between previous self-harm and subsequent self-harming behaviors, including recurrent overdose and repeated suicide attempts (27, 43–45). This association may be interpreted within a broader psychosocial context. Prior self-harm has been linked to persistent emotional distress and emotion regulation difficulties, and it frequently co-occurs with ongoing psychosocial adversity. These vulnerabilities may contribute to higher susceptibility to repeat self-harm when individuals face new stressors or unresolved life difficulties (25, 46–48). Although psychiatric history did not remain independently associated with repeated IDO in the multivariable analysis, this factor has been widely documented in the literature as being related to self-harm behaviors (49–52). The lack of an independent association in the final model may reflect overlap and interrelationships among psychosocial variables, particularly given that prior self-harm frequently co-occurs with underlying mental health disorders. This attenuation after adjustment suggests that the effect of psychiatric history on repetition risk may be mediated or confounded by prior self-harm. These findings underscore the multifactorial nature of repeated IDO, in which psychological, behavioral, and social factors interact rather than exert isolated effects. From an emergency nursing perspective, the observed association between prior self-harm and repeated IDO highlights the importance of comprehensive psychosocial assessment during emergency encounters. Routine inquiry into past self-harm experiences may support more informed clinical judgment and facilitate timely referral to appropriate mental health and follow-up services, without relying on assumptions based solely on demographic characteristics.

It should be noted that the proportion of repeated IDO observed in the present study reflects a conservatively defined, time-bound outcome rather than lifetime repetition prevalence. In this study, repeat IDO was operationally defined as a subsequent emergency department presentation for intentional drug overdose occurring within the study period, whereas lifetime history of self-harm—including prior overdose—was analyzed as a separate construct. This distinction may partly account for differences in reported repetition rates across studies and underscores the importance of clearly defined outcome measures when interpreting and comparing findings related to recurrent self-harm. Information on illicit drug use was not systematically available in this retrospective dataset and should be explored in future studies with more detailed substance use assessment.

### 4.5 Implications for emergency nursing practice

In clinical nursing practice, emergency nurses should routinely assess patients presenting with IDO for any history of previous self-harm and incorporate this information into comprehensive psychosocial assessment. As demonstrated in this study, individuals with a history of self-harm are more likely to experience repeated IDO, underscoring the importance of systematic inquiry during triage and initial nursing evaluation. In addition, age-related patterns observed in this study suggest that adolescents and young patients with a history of self-harm warrant particular clinical attention. Early identification of psychological distress during triage and initial assessment, supported by brief and feasible screening approaches, may facilitate timely recognition of patients who require further mental health evaluation. Nursing care should encompass psychological crisis assessment, empathetic communication, and appropriate family education to support coping and engagement with follow-up care. At the level of nursing management, strengthening coordination between emergency departments, psychiatric services, and community-based care is essential to improve continuity following discharge. Clearly defined nursing roles in referral, follow-up communication, and health education may help reduce loss to follow-up and support sustained engagement with mental health services. From a public health perspective, nurses can also contribute to harm reduction by promoting safe medication storage and appropriate pesticide management, particularly in community and rural settings where access to potentially harmful substances may be less regulated. These efforts align with the broader role of nursing in injury prevention and mental health promotion.

### 4.6 Strengths and limitations

This study utilized real-world data from 601 emergency department patients with IDO, providing valuable clinical insights and nursing implications as a relatively large hospital-based epidemiological investigation of IDO in China. Several limitations should be acknowledged. First, the single-center, retrospective design may introduce geographic and sampling bias and limit the generalizability of the findings. Second, the lack of detailed psychosocial variables restricted a more comprehensive examination of mechanisms underlying repeated IDO risk. Illicit drug use was not documented in the available medical records; however, because such information was not systematically assessed, its presence cannot be excluded. Third, the relatively small number of patients with repeated IDO may have affected the stability of multivariable analyses. Finally, the retrospective nature of the study precludes causal inference. Future multicenter, prospective studies are therefore needed to validate and extend these findings.

Future research should incorporate broader sociodemographic and psychosocial factors, such as family structure and only-child status, to better elucidate interactions between demographic characteristics and psychosocial vulnerability. The integration of standardized psychological assessments ((e.g., Self-Rating Depression Scale, Difficulties in Emotion Regulation Scale, and Mental Pain Tolerance Scale) may further enhance understanding of psychological mechanisms associated with IDO and recurrent IDO. In addition, mixed-methods approaches combining quantitative and qualitative data could provide deeper insight into self-harm motivations, emotional experiences, and barriers to help-seeking.

## 5 Conclusion

In summary, patients presenting with IDO were predominantly adolescents and young adults, with a higher proportion of females, and overall incidence demonstrated seasonal peaks in autumn and spring, largely driven by younger age groups. Most cases involved medication-related poisoning. A history of prior self-harm was the only variable independently associated with repeated IDO in the multivariable model. These findings underscore the importance of routine assessment of self-harm history during emergency nursing evaluations, as well as the provision of timely psychological support and family-centered education, particularly for high-risk populations such as adolescents and young adults.

At the level of nursing management, strengthening integrated and continuous care pathways linking emergency departments, psychiatric services, and community-based follow-up is essential to ensure continuity of care after discharge. From a public health perspective, promoting safe medication storage, reinforcing pesticide regulation, and enhancing collaboration between emergency and community mental health services may help reduce access to high-risk substances and support preventive care. Overall, the findings provide an evidence-based foundation for improving risk assessment and nursing interventions for repeated IDO in emergency settings and offer practical guidance for future clinical practice and service development.

## Data Availability

The data analyzed in this study is subject to the following licenses/restrictions: The dataset is not publicly available due to institutional regulations and ethical restrictions related to patient privacy. Data access requests must be reviewed and approved by the institutional ethics committee. Requests to access these datasets should be directed to shumin rong 694906176@qq.com.
