# Supplementary material for: Demographic characteristics and repeat overdose risk factors in 601 patients with intentional drug overdose in the emergency department: a cross-sectional study
Source: Front Psychiatry. 2026 Feb 5;17:1763816. doi: 10.3389/fpsyt.2026.1763816 (PMC12916651; doi:10.3389/fpsyt.2026.1763816)
Supplement: Supplementary file 1 [file Table1.docx]

Supplementary Table S1. Distribution of medication categories involved in intentional drug overdose according to ATC classification

| **ATC main group** | **Medication category** | **n (%)** |
| --- | --- | --- |
| N05A | Antipsychotics | 178 (29.6) |
| N05C | Sedatives and hypnotics | 155 (25.8) |
| N06A | Antidepressants | 140(23.3) |
| N03A | Antiepileptics | 67(11.1) |
| J01 | Antibacterials | 44(7.3) |
| M01A | Non-steroidal anti-inflammatory drugs (NSAIDs) | 26(4.3) |
| C | Cardiovascular drugs | 19(3.2) |
| R05 | Cough suppressants | 12(2.0) |
| A | Alimentary tract & metabolism | 10(1.7) |

**Note:**Percentages are calculated based on the total study population (n = 601). Percentages may not sum to 100% because some patients ingested more than one medication.
